# A Dual Marker for Monitoring MDR-TB Treatment: Host-Derived miRNAs and *M. tuberculosis*-Derived RNA Sequences in Serum

**DOI:** 10.3389/fimmu.2021.760468

**Published:** 2021-11-04

**Authors:** Claudia Carranza, María Teresa Herrera, Silvia Guzmán-Beltrán, Manuel Guadalupe Salgado-Cantú, Ivan Salido-Guadarrama, Elizabeth Santiago, Leslie Chávez-Galán, Luis Horacio Gutiérrez-González, Yolanda González

**Affiliations:** ^1^Laboratorio de Inmunobiología de la Tuberculosis, Instituto Nacional de Enfermedades Respiratorias Ismael Cosío Villegas, Mexico, Mexico; ^2^Department of Microbiology, National Institute for Respiratory Diseases Ismael Cosío Villegas, Mexico, Mexico; ^3^Laboratorio de Biología Computacional, Instituto Nacional de Enfermedades Respiratorias Ismael Cosío Villegas, Mexico, Mexico; ^4^Laboratory of Integrative Immunology, Instituto Nacional de Enfermedades Respiratorias Ismael Cosío Villegas, Mexico, Mexico; ^5^Department of Virology and Mycology, Instituto Nacional de Enfermedades Respiratorias Ismael Cosío Villegas, Mexico, Mexico

**Keywords:** drug resistance to TB, miRNAs, exosomes, *M. tuberculosis*-RNA, monitoring treatment

## Abstract

**Background:**

In the absence of a late marker of treatment failure or relapse in MDR-TB patients, biomarkers based on host-miRNAs coupled with *M. tuberculosis*-RNAs evaluated in extracellular vesicles (EVs) are an alternative follow-up for MDR-TB disease. Characterization of EVs cargo to identify differentially expressed miRNAs before and after treatment, and to identify *M. tuberculosis*-derived RNA in serum EVs from resistant TB patients.

**Methods:**

EVs were isolated from serum of 26 drug-resistant TB (DR-TB) patients and 16 healthy subjects. Differential expression of miRNAs in pooled exosomes from both untreated and treated patients was assessed and individually validated at different time points during treatment. In addition, *M. tuberculosis* RNA was amplified in the same samples by qPCR.

**Results:**

A multivariate analysis using miR-let-7e-5p, -197-3p and -223-3p were found to be a more sensitive discriminator between healthy individuals and those with TB for both DR-TB (AUC= 0.96, 95%, CI=0.907-1) and MDR-TB groups (AUC= 0.95, 95%, CI= 0.89-1). Upregulation of miR-let-7e-5p were observed at the time of *M. tuberculosis* negative culture T(3-5) for MDR-TB group or for long-term T(9-15) for MDR-TB group without diabetes (T2DM). A second pathogen-based marker based on 30kDa and 5KST sequences was detected in 33% of the MDR-TB patients after the intensive phase of treatment. The miR-let7e-5p is a candidate biomarker for long-term monitoring of treatment for the group of MDR-TB without T2DM. A dual marker of host-derived miR-let7e-5p and *M. tuberculosis*-derived RNA for monitoring-TB treatment based in serum EVs.

**Conclusion:**

A dual marker consisting of host-derived miR-let7e-5p and *M. tuberculosis*-derived RNA, could be an indicator of treatment failure or relapse time after treatment was completed.

## Introduction

Tuberculosis (TB) is a transmissible disease which still is one of the top ten causes of death worldwide. Only in 2019, between 8.9 and 11 million lived with the active TB, and 2.4 million people died from it. One quarter of the world population could already be infected with Mycobacterium tuberculosis (*M. tuberculosis*) and, therefore, at risk of developing the active form of the disease (1).

The risk of TB reactivation increases significantly in people with risk factors such as diabetes mellitus (DM), human immunodeficiency virus (HIV) coinfection, and many other conditions associated with immunocompromise (1).

Currently, treatment of drug-sensitive TB consists of a six-month regimen based on four drugs. However, drug-resistant TB (DR-TB) is defined when a person has drug resistant TB for at least one of the main TB drugs, and multi drug resistant TB (MDR-TB) is defined when a person has drug resistance to rifampicin and isoniazid, the two most efficacious first-line anti-tuberculosis drugs. Both types of drug resistant TB remain as significant challenges and major contributors to mortality. According to the same report from WHO, TB-DR killed 465,000 people in 2019 (1). Drug resistance is more probable in patients non-compliance to treatment or a primary infection with a drug-resistant strain (DR-TB) (2, 3). Treatment for multi-drug resistant tuberculosis (MDR-TB) treatment is longer (two years), more toxic and much less successful (57% cure rate) (1).

Most diagnostic tests for TB are based on either bacteriological culture or polymerase-chain reactions (PCR). Traditionally, sputum smear microscopy has been the most widely used test. Despite its high specificity, its diagnostic value is limited by its very low sensitivity. Cultures, on the other hand, can take up to 42 days to reveal detectable growth (4, 5). The Xpert MTB/RIF and the ultra-version tests detect genetic material from *M. tuberculosis*, with a sensitivity similar to microbial cultures (6). These tests yield quick results but are costly and not widely available (7).

All these diagnostic assays require a positive culture for *M. tuberculosis*. However, sputum samples from individuals with active TB, especially those with type 2 diabetes mellitus (T2DM), often fail to yield bacteria. In these cases, invasive procedures are often needed for diagnosis (8). Thus, sensitive, and specific diagnostic tests that can spare patients from more invasive procedures are warranted.

A negative sputum culture is generally considered a marker of treatment success. Nevertheless, some patients develop recurrent TB despite negative sputum cultures, which may indicate the presence of viable but nonculturable bacteria. Therefore, a reliable marker of recovery or recurrence is clearly needed (9). In many diseases, including TB, biomarkers could be useful in the diagnosis of the disease, in predicting the development of an active form and in the assessment of response to treatment or vaccination (6, 10).

While the biomarkers used for diseases are molecules produced by the host cells, the TB markers consist of mycobacterial antigens, metabolites, transcription signals and proteins that are secreted from *M. tuberculosis* in replicative status (2). Both markers may be found in extracellular vesicles (EVs) secreted by infected cells, which in turn contain microvesicles of about 30 to 100 nm. Microvesicles or exosomes have a complex composition depending on the cell of origin and may be found in body fluids like blood or serum. It has been shown that exosomes secreted by cells are essential for intercellular communication, since they can carry proteins, RNA, and DNA (11–13).

Exosomes released from infected cells can transmit signals and transfer molecules to recipient cells to trigger changes in their physiology (14, 15). In particular, microRNAs (miRNAs) are transported by exosomes which are involved in the pathogenesis of several respiratory diseases, including TB, in which they play a relevant role in pulmonary inflammation and pathogenesis (14, 15). The expression of miRNA varies across different pathological conditions, which suggests that microvesicles may also reflect disease status (16).

It has been observed that *M. tuberculosis*-infected macrophages can secrete miRNAs within exosomes. *In vitro* studies have shown that monocyte-derived macrophages infected by *Mycobacterium bovis* BCG secrete exosomes containing miR-1224, -1293, -425, -4467, -4732, -484, -5094, -6848, -6849, -4488, and -96 (17).

By the same token, exosomes miRNAs of individuals with TB infection have been compared with those healthy volunteers (18). The miR-1246, -2110, -370-3P, -28-3p and -193b-5p are expressed in those with active TB. Furthermore, exosomes may contain mycobacterial miRNA as well as highly antigenic mycobacterial proteins such as ESAT-6 (Rv3875), the Ag85 complex (Rv3804c, Rv1886c, and Rv0129c), MPT64 (1980c), and MPT63 (1926c) (19).

Since there seems to be a TB-specific pattern of exosomes miRNA secretion, it follows that these molecules can be potential biomarkers of DR-TB. To prove this, more studies are needed to evaluate exosomes miRNAs in actual patients with DR-TB. Knowledge generated from such studies could help in the diagnosis, prognosis, and treatment of the disease. Thereby, in this study we identify the spectrum of miRNAs generated in host’s lung tissue in response to *M. tuberculosis* and sequences from mycobacteria that circulate within exosomes. This will help us to identify those markers associated to the pathogen as well as those associated with the host.

## Methods

### Study Participants

Twenty-six patients with DR-TB were studied between 2010 and 2018. DR-TB group included those with rifampicin resistant (RR), multi-drug resistant (MDR), and extensively drug-resistant (XDR) strains; all patients had a confirmed diagnosis of TB and were under Directly Observed Therapy (DOT). Patients were followed for 15 months at the Instituto Nacional de Enfermedades Respiratorias (INER) in Mexico City. A blood sample was taken at each follow-up visit. This sample was centrifuged to obtain serum and stored at -80°C. Additionally, 16 healthy volunteers were included as control group and their samples underwent the same procedure. An informed consent was obtained from all participants. The use of these samples was approved by the Institutional Review Board. Written consent was obtained from all participants, and the Ethics Committee approved this study (No. C57-17).

### Biological Samples Tested

We analyzed the samples from 26 TB patients with drug resistant TB diagnoses (21 MDR, three RR and two XDR); the blood samples were taken at: T(0), untreated patients; T(3-5), one sample between 3 to 5 months after treatment; T(6-8), one sample between 6 to 8 months after treatment, and T(9-15), one sample between 9 to 15 months after treatment. A total of four samples per patient were processed. The samples were classified into four categories: 1) those from DR-TB patients exhibiting any resistance types (RR, MDR or XDR); 2) those from MDR-TB patients; 3) those from MDR-TB patients without T2DM (MDR-TB-T2DM−, and 4) those from MDR-TB patients with T2DM (MDR-TB-T2DM+).

### Exosomes Extraction From Serum

Exosomes were purified from the serum using the Exo-Quick™ exosome precipitation solution (System Biosciences, USA), following the instructions of the manufacturer, and adding a filtration procedure step with a 0.22 μm filter before the procedure. In brief, filter serum was centrifuged at 3000 g and ExoQuick™ exosome precipitation solution was added and incubated, then it was centrifuged at 1500 g and the pellet was resuspended in 200 μl of PBS. Exosomes or EVs were obtained from 300 μl of serum to perform DNA and RNA extraction, protein extraction and miRNA analysis. EVs were stored at -20°C until further processing.

### Extraction and Quantification of Exosomes Proteins

Protein was obtained from 100 μl of exosomes using buffer RIPA (cat. 89901, Fisher Scientific, USA.) plus a protease inhibitor cocktail (cat. 78430, Thermo Fisher Scientific, USA). The mix was sonicated for 15 seconds for total lysis and then centrifuged at 14000 g for 15 minutes at 4°C. The supernatant was recovered, and total protein was quantified using the Quick Start assay (cat. 500-0202, Bio-Rad, USA). The absorbance was read at 595 nm (Epoch™, Bioteck) and the protein quantitation was calculated by standard curve.

### SDS-PAGE-Western Blot Assay for Exosomal Tetraspanin and Exosomal *M. tuberculosis*-Derivate Proteins

**Figure 1:**
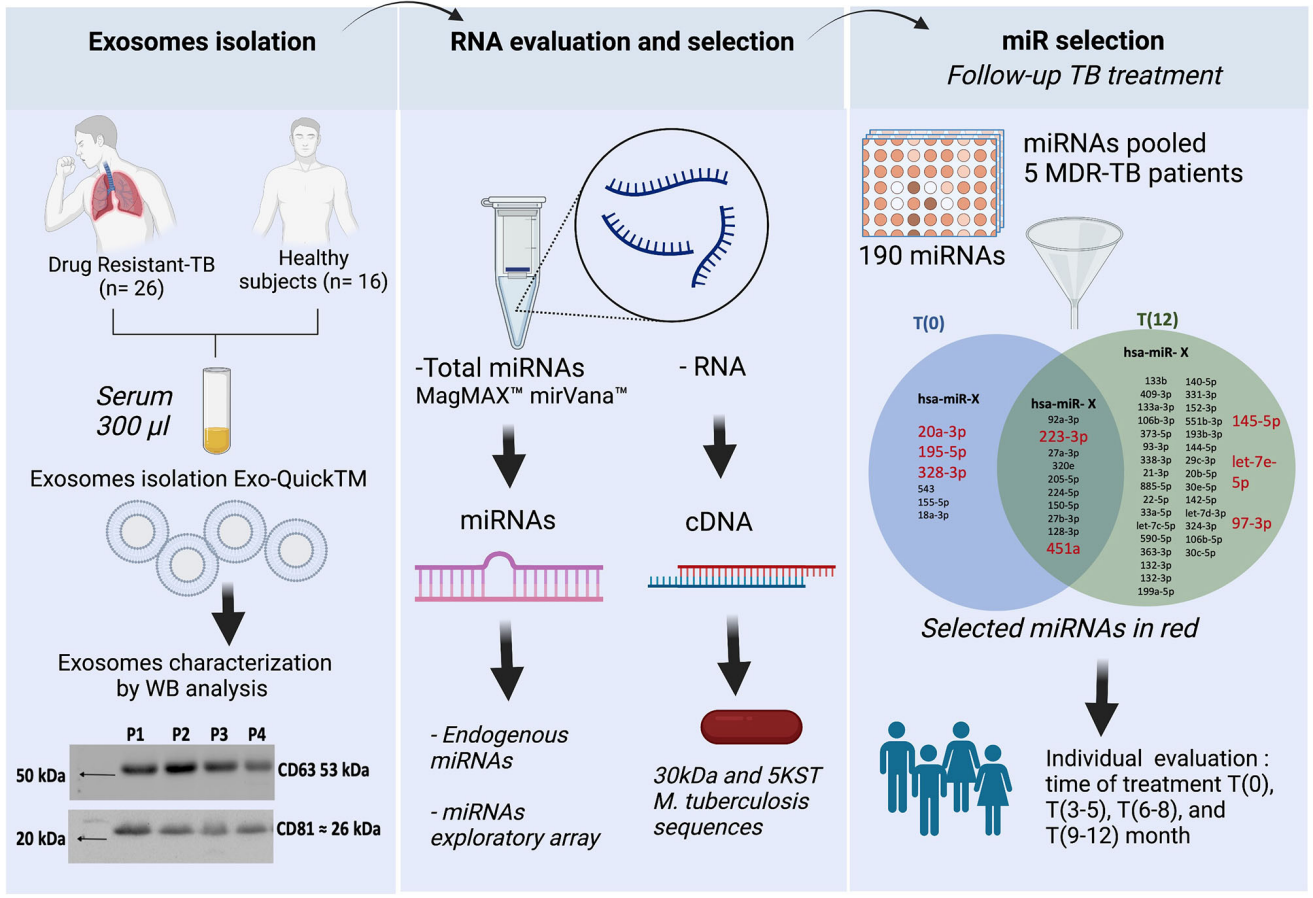
Workflow chart. General strategy in three steps. First step, the exosomes isolation and characterization showed CD63 and CD81 tetraspanin expression in serum exosomes from DR-TB patients. Second step, extraction of both host-miRNAs and *M. tuberculosis*-RNA. Third step, miRNAs differentially expressed in serum exosomes from MDR-TB patients at T(0) and T(12). In red, the 8 miRNAs selected for individual analysis. BioRender, publication and licensing rights (QX22QHOTQ4).

The EVs total protein was mixed with Laemmli buffer (cat.1610747, Bio-Rad, USA) with β-mercaptoethanol (Bio-Rad, USA). Then, proteins were separated by SDS-polyacrylamide gel and transferred to a nitrocellulose membrane Immobilon-P (MilliporeSigma, USA). The membranes were incubated with anti-CD63 (ab216130, Abcam, UK), -CD9, -CD81, -hsp70 ExoAb Antibody Kit (cat. EXOAB kit, System Biosciences, USA), ESAT-6 (ab26246, Abcam, U.K), -CFP-10 (PA1-19445, Thermo Fisher, USA), -Ag-38kDa (NB100-62769, Novus Biological, USA), -Ag-85B (ab43019, Abcam, U.K), and -MPT64 (CSB-PA14947A0Rb, Cusabio, USA), and then with anti-IgG-HRP (cat. EXOAB kit) or anti-IgG-HRP (65-6120, Thermo Fisher, USA). The chemiluminescent substrate Clarity (cat. 170-5060, Bio-Rad, CA, USA) was added, and immunocomplexes were detected using system ChemiDocMP Imaging (Bio-Rad, Hercules, USA). CD63 and CD9 tatraspanins expression is shown in the workflow chart (**Figure 1**).

### Extraction of Total Small RNAs and miRNA Retrotranscription From Serum Exosomes

The RNA was obtained with the MagMAX™ *mir*Vana™ Total RNA Isolation Kit (cat. A27828, Applied Biosystems-Thermo Fisher Scientific, USA), which was used as per the instructions of the manufacturer. The total RNA of exosomes was recovered and stored at -20°C until further use.

For RNA synthesis of complementary DNA was used the TaqMan™ Advanced miRNA cDNA Synthesis Kit (cat. A28007, Applied Biosystems-Thermo Fisher Scientific, USA) following the manufacturer instructions. RNA-samples were thawed and underwent three procedures in sequence: first a polyadenylation of miRNA catalyzed by one poliA polymerase, then an adaptive ligation and finally a retrotranscription by an inverse transcriptase. The complementary DNA (cDNA) was synthetized in the Step One Plus instrument (Applied Biosystems, Thermo Fisher Scientific, USA), according to manufacturer instructions and stored at -20°C until further use.

### Evaluation of Endogenous miRNAs Expression From Exploratory Array

The miRNA Human Endogenous Controls 96-well Plate TaqMan™ Advanced (cat. A34642, Applied Biosystems-Thermo Fisher Scientific, USA) was used to evaluate 30 endogenous control miRNAs, including two exogenous (non-human) controls for normalization of sample input. Briefly, PCR was performed mix the TaqMan Fast Advanced Master Mix (cat. A44360 Applied Biosystems-Thermo Fisher Scientific, USA) and the pool of cDNA from five MDR-TB patients before treatment (T0) or after 12 months of treatment (T12) and the mix was added to plates. The qPCR was run in the Step One Plus instrument according to manufacturer instructions. The data are shown in the **Supplementary File**.

### Evaluation of Exosome miRNAs Expression From Exploratory Array and Individual Validation

We used pre-spotted TaqMan Advanced miRNA assays in a 96-well fast plate (cat. A31813 Applied Biosystems, Thermo Fisher Scientific, USA). This kit contains two plates for 188 unique miRNAs for serum/plasma samples, 2 exogenous controls (cel-miR-39-3p, ath-miR159a) and an endogenous control (hsa-miR-16-5p) for normalization of data results. Briefly, PCR was performed by mixing the TaqMan Fast Advanced Master Mix solution and the pool of cDNA from five MDR-TB patients at T(0) or T(12), and added to plates. The qPCR was run in the Step One Plus instrument, according to manufacturer instructions. The differentially expressed miRNAs in the pool at T(0) *vs* T(12) are shown in the workflow (**Figure 1**) and in the **Supplementary File**.

For individual validation, we selected eight miRNAs: two endogenously expressed at C_T_<25, three miRNAs expressed at T(0) and down-expressing after 12 months with C_T_<32, and three miRNAs not expressed at T(0) and up-expressed at T(12) with C_T_<32. Briefly, cDNA for individual miRNAs expression was evaluated at four different times: T(0), T (3-5), T(5-8) and T(9-15), when it was mixed with the TaqMan Fast Advanced Master Mix and the specific probe for microRNA assays (Custom TaqMan^®^. Applied Biosystems, ThermoFisher Scientific, CA, USA), according to the below table.

**Table:** 

miRNA name	Target sequence
hsa-miR-20a-3p	ACUGCAUUAUGAGCACUUAAAG
hsa-miR-195-5p	UAGCAGCACAGAAAUAUUGGC
hsa-miR-328-3p	CUGGCCCUCUCUGCCCUUCCGU
hsa-miR-145-5p	GUCCAGUUUUCCCAGGAAUCCCU
hsa-let-7e-5p	UGAGGUAGGAGGUUGUAUAGUU
hsa-miR-197-3p	UUCACCACCUUCUCCACCCAGC
hsa-miR-223-3p	UGUCAGUUUGUCAAAUACCCCA
hsa-miR-451a	AAACCGUUACCAUUACUGAGUU

The qPCR was run in the Step One Plus instrument, according to manufacturer instructions. The C_T_ values of individual miRNAs are shown in the **Supplementary File**.

### DNA and Total RNA Exosome Extraction and *M. tuberculosis* Sequences Amplification by qPCR

The DNA was isolated with the Exosomal DNA Extraction Kit (101Bio, USA) according to the manufacturer instructions. RNA was extracted with the total exosome RNA and protein isolation kit (Invitrogen-ThermoFisher Scientific, USA) according to the manufacturer instructions. Then, the complementary DNA (cDNA) was synthetized using RNA as template and the TaqMan™ MicroRNA Reverse Transcription kit (cat. 4366597, Applied Biosystem-Thermo Fisher Scientific, USA) with hexamers (cat. N8080127, Invitrogen, Thermo Fisher Scientific Inc., USA), and was amplified using a thermocycler Verity (Applied Biosystems-ThermoFisher Scientific, USA)

Finally, mycobacterial sequences were evaluated by quantitative real time PCR (qPCR), using exosomal cDNA, Maxima SYBR™ Green/ROX qPCR Master Mix (Thermo Scientific Inc., USA), and the specific forward/reverse primers for *M. tuberculosis* antigens.

**Table:** 

Antigen	Primers 5´- 3´
MPT64	FW: GTGAACTGAGCAAGCAGACCG RV: GTTCTGATAATTCACCGGGTCC
MPB64	FW: GACTTCTGGTCGGGGTAGTAAC RV: CTGTCGTTTTGCTCTGTTGTTC
5KST	FW: TTGCTGAACTTGACCTGCCCGTA RV: GCGTCTCTGCCTTCCTCCGAT
19 kDA	FW: GAGACCACGACCGCGGCAGG RV: AATGCCGGTCGCCGCCCCGCCGAT
30 kDa	FW: TGTACCAGTCGCTGTAGAAG RV: GACATCAAGGTTCAGTTCC
CFP-10	FW: AGGTAATTTCGAGCGGAT RV: CACTGGCCCTGCAACGAA
ESAT-6	FW: AAGCTCGCAGCGGCCTGG RV: CCTGACCGGCTTCGCTGA
Ag85c	FW: AAGGTCCAGTTCCAGGGCG RV: ATTGGCCGCCCACGGGCATGAT
16S rRNA	FW: GCCGTAAACGGTGGGTACTA RV: TGCATGTCAAACCCAGGTAA

The DNA from *M. tuberculosis* H37Rv strain was included as positive control and water for PCR was used as a negative control. In the samples, a result was considered positive when the melting temperature was comparable to the positive control. Then, we visualized the amplicons in agarose gel electrophoresis (1.8%) with GelRed (cat. 41003, Bioutum) and documented by the ChemiDocMP Imaging System.

### Statistical Analysis

Raw C_T_ data were normalized using the Delta C_T_ method (δC_T_). To that end, average expression stability (defined as M-value) of the reference miRNA was determined using the GeNorm algorithm (20). Briefly, this method iteratively compares the mean expression value of tested miRNAs and selects those with lowest M-value. Then, for each sample we subtracted the C_T_ value of the most stable miRNA from the C_T_ value of the remaining miRNAs.

Expression differences between cases and controls were assessed by an unpaired two-samples Mann-Whitney test. To evaluate changes in expression that are associated to treatment, expression at different endpoints (i.e., T(3-5), T(6-9) and T(9-15) months) were compared against expression at T(0). Significance was evaluated with a Wilcoxon signed-rank test and a p-value < 0.05 was considered significant. Receiver Operating Characteristics (ROC) analysis and multiple regression were performed with the pROC package and the glm function in R, respectively. The evaluation of transitions was based on the δC_T_ value between all the possible time points for all samples of study. The analysis was done using the Matlab R2017b software.

## Results

### Characteristics of Study Groups

The patients with drug resistant TB included patients with rifampicin resistant (RR), multi-drug resistant (MDR), and extensively drug-resistant (XDR) strains; all patients had a confirmed diagnosis of TB and were under Directly Observed Therapy (DOT). The 14% of patients were underweight. The main comorbidity was type 2 diabetes mellitus (T2DM), 33% of patients have it. Sixteen age- and sex-matched healthy subjects were included for miRNAs expression analysis (**Table 1**).

**Table 1:** Characteristics of the study participants.

	*Patients (n=26)*	*Healthy controls (n=15)*	*P value*
Age, yr	43.5 (19–77)	45 (27–61)	0.6859
Sex, male (%)	18 (69.2)	10 (66.6)	>0.9999
BMI	23.2 (17-27)	26 (23-33)	0.0050
**Comorbidities**			
Diabetes (%)	9 (34.5)	–	–
Alcoholism (%)	9 (34.5)	–	–
Smoking (%)	2 (7.7)	–	–
Malnutrition (%)	15 (5.77)	–	–
**Pulmonary Tuberculosis**			
RR/MDR-TB (%)	3 (15.4)	–	–
MDR-TB (%)	21 (73)	–	–
XDR-TB (%)**TB Treatment**	2 (7.7)	–	–
Individualized *(%)	9 (42.8)	–	–
**Clinical data**			
LeukocytesWBC count, x10^3^/mm^3^	9.7 (4.3–18.8)		
Neutrophil count, x10^3^/mm^3^	7.4 (1.7-17.3)		
Lymphocyte count, x10^3^/mm^3^	1.6 (0.9–2.4)		
Hematocrit %	41.2 (27.2-52.8)		
Hemoglobin g/ml	13.6 (9.4-17.7)		
NLR ratio	4.8 (0.9-18.8)		
**Metabolic characteristics**			
HbA1c % T2DM	9.2 (5.5-13.5)		
Glucose, mg/dl	99 (75-525)		
**Blood Chemistry Profile**			
Creatinine, mg/dl	0.76 (0.51-1.1)		
BUN, mg/dl	10 (5.8-20)		
Albumin, mg/dl	3.6 (2.3-4.4)		
Urea, mg/dl	22 (12.4-42)		
**Renal function**MDRD ml/min/1.73 m^2^	109.3 (67-7167.3)		
CKD/EPI ml/min	104 (64-134)		
CrCL ml/min	102.3 (49-209.2)		
Nephrotoxicity	17.4%		
Hearing damage	35%		

*Individualized include the drugs: standard antibiotics and others such as amoxicillin, clavulanic acid, ceftriaxone, linezolid,

clofazimine and dalamanid. BMI, Body Mass Index; RR/MDR-TB, Rifampicin-Resistant-MDR TB; MDR-TB,

Multidrug-resistant TB; XDR-TB, Extensively drug-resistant TB; WBC, White Blood Cell; NLR,

Neutrophil-Lymphocyte Ratio; HbA1c, hemoglobin A1c; BUN, Blood Urea Nitrogen; MDRD,

Modification of Diet in Renal Disease; CKD/EPI, Chronic Kidney Disease Epidemiology Collaboration;

CrCL, Creatinine clearance rate.

### Forty miRNAs Differentially Expressed Before and at 12-Month Follow-Up Treatment Were Identified in the Pool of Exosomal miRNAs

There are no constitutive genes for the evaluation of miRNAs; therefore, endogenous genes are used to perform normalization of miRNA expression. The analysis of 32 endogenous miRNAs in a pool of serum exosomes from five MDR-TB patients comparing T(0) *vs* T(12) did not show any stable endogenous miRNAs at any of these time points (**Supplementary File**). However, the analysis of the expression of an array of 180 miRNAs in the same pool of miRNAs showed 10 stable miRNAs in the serum exosome pool at T(0) and (T12), and we selected two of them to normalize the data. Additionally, we identified 40 miRNAs up- or down-expressed at T(0) *vs* T(12). Six were up-expressed at T(0) and 34 were up-expressed at T(12) (**Figure 1**).

Of the 40 miRNAs, we analyzed eight individually: miR-223-3p and -451a, -20a-3p, -195-5p and -328-5p, -145-5p, -197-3p and -let7e-5p. We identified miR145-5p as the miRNA that is more consistently expressed during TB treatment. Therefore, we used it to calculate the ΔC_T_. The miRNAs expression from individual serum exosomes are shown in the **Supplementary File**.

### Differential Expression of miR-197-3p, miR-223-3p and miR-let7e-5p in DR-TB Disease

The molecular testing shows 69% sensitivity for smear-negative, culture-positive specimina (21). Since some patients with suspected TB cannot be diagnosed through these methods, here we analyzed miRNA expression associated to DR-TB diagnosis.

**Figure 2:**
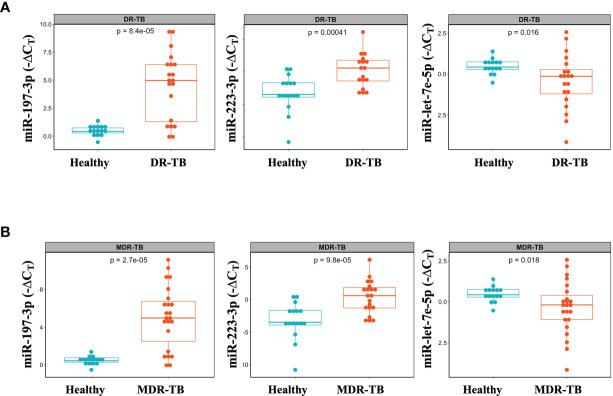
Differential miR-let-7e-5p, miR-197-3p and miR-223-3p expression in serum exosomes from DR-TB patients and healthy subjects. Serum exosomes were isolated, miRNAs amplification was done by qPCR. The data were expressed as ΔC_T_, defined as the relative expression of each miRNA with respect to the endogenous control (mirR-145-5p). Box plots depict medians and quartiles. **(A)** DR-TB patients (n = 24) and **(B)** MDR-TB patients (n = 20) and healthy subjects (n = 16), p < 0.05, (TB *vs.* Control), DR, Drug Resistant; MDR, Multi Drug Resistant. Raw C_T_ data were normalized, and expression differences between cases and controls were assessed by an unpaired two-samples Mann-Whitney test. Only statistical differences with p < 0.05 are shown.

We identified three miRNAs associated to DR-TB disease: miR-197-3p and -223-3p, which are downregulated, and miR-let7e-5p, which is upregulated in DR-TB patients compared to healthy subjects (**Figure 2A**). Since the most represented group was that of MDR-TB, we focused our search for differential miRNA expressions in this group, only to find that the same statistical differences were conserved (**Figure 2B**).

### miRNA-Model Increases the Sensitivity and Specificity for TB Disease

**Figure 3:**
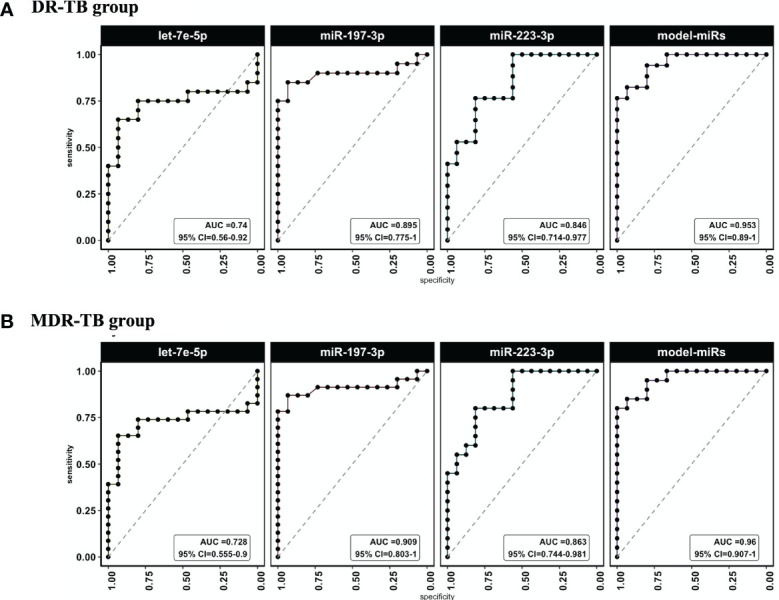
ROC curves and AUC values for miRlet-7e-5p, -197-3p and -223-3p miRNAs in TB patients *vs* healthy subjects. ROC analysis was done to evaluate sensitivity and specificity. ROC curves were used to compare the relative expression of the three miRNAs in DR-TB and MDR-TB patients with those expressed in healthy subjects. **(A)** AUC and CI to miR-let-7e-5p, -197-3p and -223-3p for DR-TB patients and **(B)** (AUC) and CI to miR-let-7e-5p, -197-3p and -223-3p for MDR-TB patients. AUC, area under the curve; CI, confidence interval; ROC, receiver operator characteristic. Only statistical differences with p < 0.05 are shown.

We calculated the sensitivity and specificity of miR-197-3p, -223-3p and -let7e-5p performing a ROC curve analysis, both as single predictors and in combination using a multivariable model to distinguish the DR-TB or MDR-TBs group from the group of samples from healthy individuals. We observed that miRNA-Model reached an Area Under the Curve value (AUC) = 0.96 (95% CI=0.907-1) for DR-TB patients (**Figure 3A** and **Table 2**) and AUC= 0.95, (95% CI= 0.89-1) for MDR-TB patients (**Figure 3B** and **Table 2**), showing to be a more sensitive discriminator between healthy and diseased individuals.

**Table 2:** ROC analysis for DR-TB or MDR-TB groups *vs* Healthy subjects.

Group	miR	AUC	AUCCI.min	AUCCI.max	Cutpoint (-ΔCt)	Sensitivity	Specificity
DR-TB	miR-197-3p	0.909	0.803	1	-0.054	0.652	0.933
	let-7e-5p	0.728	0.555	0.9	0.985	0.87	0.933
	223-3p	0.863	0.744	0.981	-1.523	0.8	0.812
	model-miRs(197-3p + let-7e-5p +223-3p)	0.96	0.907	1	0.714	0.8	1
MDR-TB	197-3p	0.895	0.775	1	-0.054	0.65	0.933
	let-7e-5p	0.74	0.56	0.92	0.985	0.85	0.933
	223-3p	0.846	0.714	0.977	-1.523	0.765	0.812
	model-miRs(197-3p + let-7e-5p +223-3p)	0.953	0.89	1	0.714	0.765	1

AUC, Area Under the Curve.

### miRlet-7e-5p and miR328-3p Expression Change at the Time of *M. tuberculosis* Negative Culture in DR or MDR TB Patients

**Figure 4:**
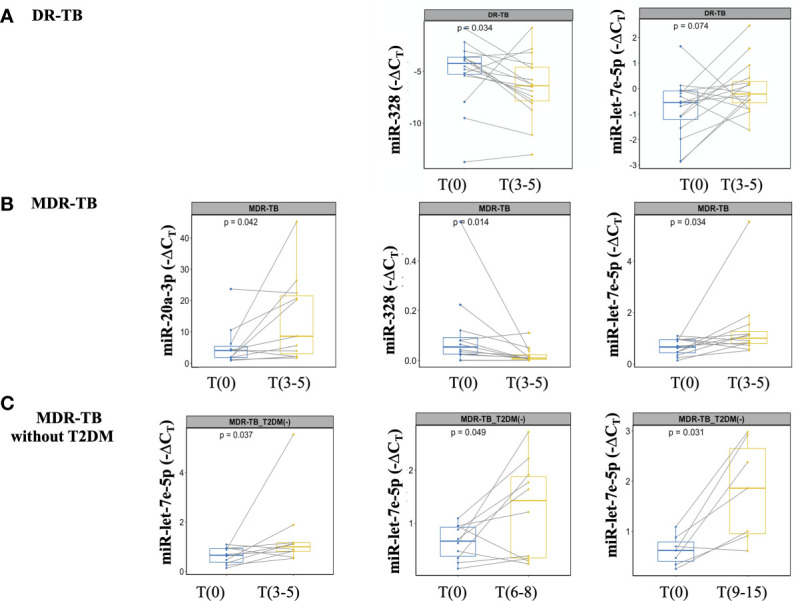
miRNAs detection at the time of *M. tuberculosis* negative culture from DR-TB patients and treatment monitoring from MDR-TB without T2DM. miRNAs expression analyzed at T(0) and T(3-5). The data are expressed as ΔC_T_, box plots depict medians and quartiles. Lines show the expression change for each patient. **(A)** DR-TB patients, **(B)** MDR-TB patients, and **(C)** MDR-TB without T2DM. Wilcoxon signed-rank test T(0) *vs.* T(3-5) or T(6-8) or T(9-15). Only statistical differences with p < 0.05 are shown.

A sputum culture-negative conversion is currently the most objective indicator of response during early stage of TB treatment (22). Thus, miRNA expression at T(3-5) months was analyzed after all patients showed negative sputum cultures. At this time point, significant changes in miR-328-3p and -let-7e-5p levels were detected: miR-328-3p was upregulated, whereas miR-let-7e-5p was downregulated (**Figure 4A**). Since some expression levels for some patients did not change the same way, an additional analysis was performed in the subgroup of samples from MDR-TB patients, where a significant difference for miR-20a-3p, -328-3p and -let-7e-5p was observed (**Figure 4B**).

### Long-Term Treatment Monitoring Was Associated With miR-let7e-5p in the Group of MDR-TB Without T2DM

Treatment monitoring should be performed at least once a month during the intensive treatment phase (first six months), and then every 1 or 2 months during the continuation phase. Delay to identify treatment failure or the emergence of resistant strains of pulmonary TB leads to severe disease and transmission (22). No differences in the expression of miR-20a-3p, -195-5p, -197-3p, -223-3p, -328-3p, -451a and -let-7e-5p at T(6-8) or T(9-15) were found between the DR and MDR groups. Given that T2DM is a metabolic disorder that can affect biomarker assessment, we performed an additional analysis excluding patients with T2DM. As shown in **Figure 4C**, miR-let7e-5p was differentially expressed at early time points T(3-5), as well as at the late time points T(6-8) and T(9-15).

### Transition Probabilities of miR-let-7e-5p Over Time Points as an Indicator of Changes

**Figure 5:**
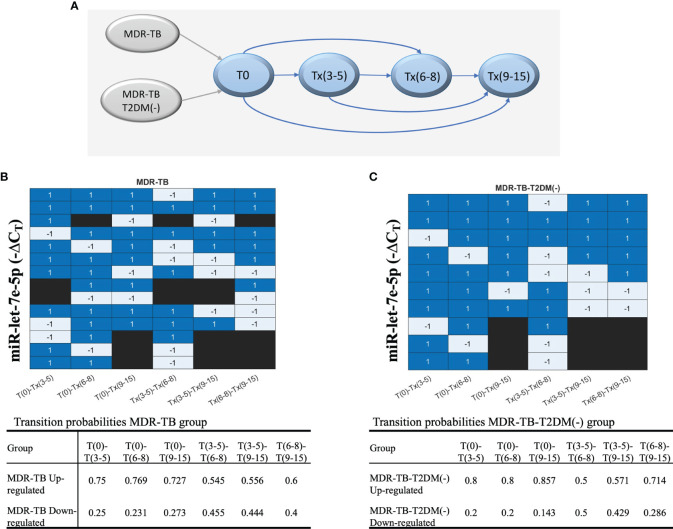
The behavior of each group of patients, showing the evolution of each patient according to the ΔC_T_ values during the periods of time. **(A)** The transitions model comparing six times. Upregulation or downregulation in the ΔC_T_ value between time points is illustrated in a heatmap. Blue: upregulation in the ΔC_T_ value in the next time point or the target time point; gray: downregulation in the ΔC_T_ Cs value, and black: no transition between time points. **(B)** MDR-TB group and **(C)** MDR-TB-T2DM (-) group. The table includes results of the transition probabilities of the two groups: MDR-TB, MDR-TB-T2DM(-). Each group is divided at the same time point into 2 subgroups according to the ΔC_T_ values of transition: upregulated or downregulated.

Since long-term identification of miRNAs could be an indicator of drug failure or relapse. an additional analysis was conducted to evaluate the transition pattern of miRNAs between timepoints. The diagram of comparisons or transitions between the timepoints (stages) is shown in **Figure 5A**. It was found that miR let-7e-5p is the only miRNA that reported an increase in δC_T_ values in most comparisons, maintaining a probability greater than 50%. The transitions of miR-let7e-5p to an upregulated or downregulated state over time are shown in a heatmap (**Figure 5B**). The probabilities for the miR let-7e-5p associated to the MDR-TB and MDR-TB-T2DM(-) groups are shown in **Figure 5C**. We found concordance between the changes observed in **Figure 5** with the transition probability analyses at the more distant T(6-8) and T(9-12) time points (**Figure 5**).

### Presence of 30kDa and 5KST RNA From *M. tuberculosis* in Serum Exosomes of DR or MDR TB Patients

Although biomarkers based on the host response to *M. tuberculosis* are useful diagnostic tools, they may be affected by new or concomitant diseases. We found that the long-term expression of miR-let-7e-5p was associated only with MDR-TB patients without DM, and that some patients did not change in the same way. Therefore, it was necessary to search for a second pathogen-based marker.

**Figure 6:**
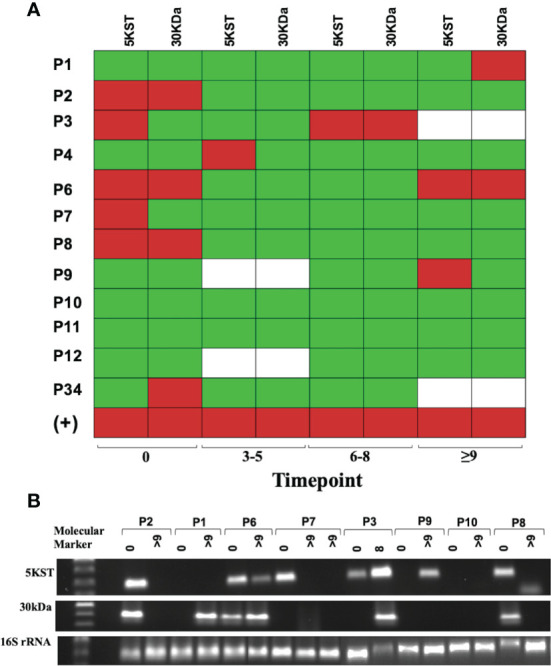
Mycobacterial 5KST and 30kDa antigens are in exosomes from DR-TB patients. Representative results from amplification fragments are presented as a heat map for **(A)** 5KST and 30kDa (n =12). Red square = positive amplification; green square = negative amplification, and white square = non-determined. **(B)** Electrophoresis of 5KST, 30kDa and 16S rRNA amplification fragments.

The presence of pathogen-derived RNA in exosomes released during macrophage infection has been reported (23). In view of the heterogeneity of the exosome cargo, we decided to evaluate the presence of proteins, DNA, and RNA sequences from TB patients. Neither DNA sequences (Mpb64, 5KST, 19kDa, CFP-10, ESAT-6, Mpt64, 30kDa and Ag85c) nor proteins (ESAT-6, CFP-10, 38kDa, Ag85b and MPT64) were found in serum exosomes from DR-TB patients (data not shown). Nevertheless, RNA sequences for 30kDa and 5KST antigens in the serum exosomes from DR-TB patients were found. **Figure 6** shows the time point of *M. tuberculosis* antigen expression and PCR products in a heat map (**Figure 6B**). Antigens were expressed before treatment in 50% of DR-TB patients. However, after the intensive phase of treatment, *M. tuberculosis* RNA sequences were detected in only 33%. These data could also suggest treatment failure or relapse.

### A Dual Marker for Monitoring MDR-TB Treatment

**Figure 7:**
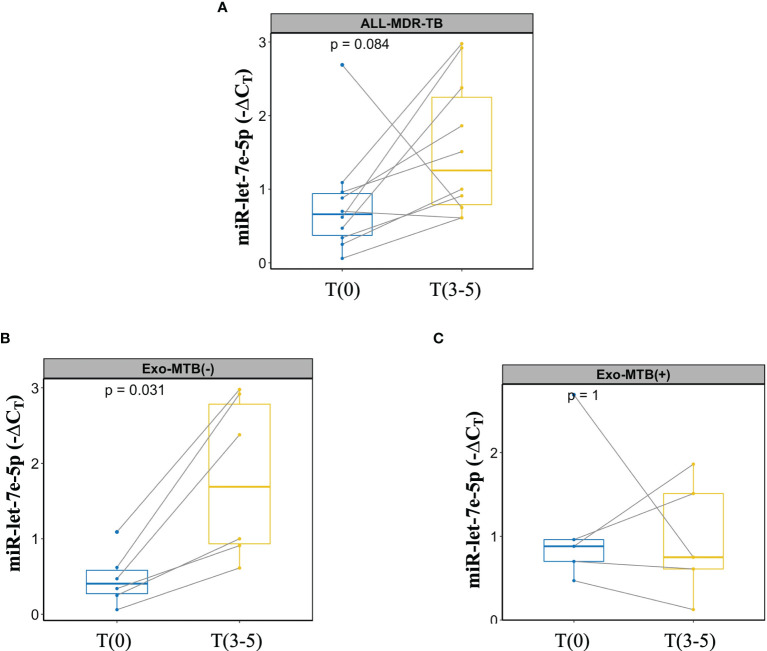
Dual marker evaluation in patients with MDR-TB. Lines show the change in expression for each patient. **(A)** miR-let7e-5p expression in MDR-TB group, **(B)** MDR-TB patients negative for 30kDa or 5KST *M. tuberculosis* sequences. **(C)** MDR-TB patients positive for 30kDa or 5KST *M. tuberculosis* sequences.

Finally, miR-let-7e-5p expression and *M. tuberculosis* antigens detected in the exosomes of MDR-TB at T(9-15) were evaluated for concordance. **Figure 7A** shows the change in miR-let7e-5p expression over time from T(9-15) in MDR-TB patients; despite the significant differences, the expression of miR-let7e-5p did not increase in some patients. Thus, the data have been disaggregated into two subgroups: one for exosomes negative to *M. tuberculosis* (**Figure 7B**), in which an increase of miR-let7e-5p is observed in all patients, and one for exosomes positive to 30kDa or 5KST proteins, in which miR-let7e-5p expression decreased in 60% of the patients (**Figure 7C**).

## Discussion

Despite recent advances in TB research, diagnosis and treatment monitoring still remain a global challenge, further exacerbated by the emergence of multidrug-resistant (MDR-TB) and extensively drug-resistant (XDR-TB) strains (24). It has been reported that *M. tuberculosis* may use exosomes as a mechanism for releasing molecules into host cells. The molecules identified are proteins, peptides and small RNA sequences (miRNAs) derived from both host and pathogen (25). Host RNAs are present in exosomes during the *M. tuberculosis* infectious process, and are potential biomarkers for the detection or diagnosis of latent and active TB (26). We use these findings to propose a dual test to detect drug failure or the emergence of resistant strains during the monitoring of treatment.

The DR-TB diagnosis is based on microbiological testing in which the sputum specimina are smeared directly onto the slides and stained using the Ziehl-Neelsen (ZN) technique; in spite of a high specificity, the sensitivity of the test has been reported to vary from 20 to 80% (21). Culture of *M. tuberculosis* is the gold standard for diagnosis and cure of TB, as it is more sensitive than microscopy; however, around 10 bacteria/ml are needed, with a sensitivity of 80–85% and a specificity of approximately 98% (27, 28). The limitation of both methods is reflected on the relatively high proportion of patients who remain undiagnosed.

In this work, we propose the use of miRNAs from serum exosomes, which are non-invasive prognostic markers, and suggest the simultaneous evaluation of the three host derivate miRNAs (-let-7e-5p, -197-3p and -223-3p) for the identification of DR-TB (including RR, MDR and XDR) in those patients for whom the *M. tuberculosis* ZN staining or culture are negative. The expression of miR-197-3p and -223-3p are increased, while miR-let-7e-5p decreases in DR and MDR-TB patients. The expression of these three miRNAs is associated with an increased likelihood of MDR-TB in a multivariate model (ROC curve AUC= 0.96 and CI of 0.907-1). To our knowledge, this specific pattern of miRNAs expression had not been previously reported. It had been observed that miR-let-7e-5p, -let-7d-5p, -450a-5p, and -140-5p were expressed among patients with LTBI (28), while miR-223-3p was decreased in plasma samples from patients with TB compared to healthy controls (29). Now we are adding to those findings the observation that the expression of miR-let7e-3p is downregulated while miR-223-3p is increased in MDR-TB patients. According to our data and those published by Lingna et al., miR-let-7e-5p is suppressed during the active phase of the TB caused for any strain of *M. tuberculosis*, while its expression is increased in the TB-latent or after one year of drug treatment for MDR-TB patients, suggesting its use as a possible biomarker for non-resistant and resistant pulmonary TB (18).

miRNAs have regulatory functions: miR-223-3p targets the gene STAT1, involved in interferon-gamma signaling during TB disease (29); miR-let-7e-5p acts as a tumor suppressor, and its down-regulation may promote the development and progression of carcinomas (30). Finally, miR-197-3p binds to the interleukin-1beta (IL-1β) receptor, contributing to autoinflammatory processes (31). Nevertheless, the role of these miRNAs in tuberculosis remains to be determined.

To date, only microbiological methods based on sputum sample are available to monitor TB treatment; however, an important limitation of these methods lies in the availability of biological samples, since patients stop expectorating a few months after starting treatment. Furthermore, even if bacilli are detected again in sputum, few changes in the therapeutic regimen can be made (22). For this reason, we propose measuring miRNAs as part of the monitoring of DR-TB treatment: if at the time of sputum conversion T(3-5), the miR-let-7e-5p expression becomes downregulated, while the miR-328-3p is up-expressed, it is likely that RR, MDR or XDR strains have appeared. Additionally, we have shown that the up-expression of a particular miRNA, the miR-20a-3p, is associated with a specific type of resistance: that of the MDR group. Thus, miR-328-3p and -let-7e-5p expression could be useful to analyze in conjunction with other clinical and microbiological data to monitor DR-TB patients during the early stages of treatment.

To summarize, to evaluate early-term treatment efficacy, we propose to determine two miRNAs (miR-let-7e-5p and -328-3p) for all DR-TB patients and three miRNAs (miR-let-7e-5p, -328-3p and -20a-3p) for MDR-TB patients. The strategy of using a set of biomarkers had been suggested previously for MDR-TB diagnosis: specifically, with a model based on five biomarkers (CD44, KNG1, and miR-4433b-5p, -424-5p, and -199b-5p) (32). However, the latter study has important differences with our own, namely, the type of sample (serum) and the tools used (liquid chromatography-tandem mass spectrometry and sequencing).

The main challenge of this study was to find a biomarker to predict treatment failure or relapse after the completion of the intensive phase (22). Although we expected to find biomarkers that could be useful for all patients regardless of their comorbidities, we did not find any miRNA expression pattern applicable to the MDR-TB group in general, but only in MDR-TB patients without T2DM. In this group, we observed that miR-328-3p and -let7e-5p correlate with the time of smear conversion T(3-5), and that miR-let7e-5p correlates with the intensive phase of treatment T(9-15). Based on these data, miR-let7e-5p expression may be a biomarker in patients with MDR-TB without T2DM.

The presence of T2DM in TB patients not only tends to make biomarker assessment more difficult. On one hand, oral antihyperglycemic drugs can alter the pharmacokinetics, safety, and clinical effects of anti-TB drugs. On the other, T2DM patients are also at a higher risk of TB treatment failure, death, and relapse after cure. Therefore, treatment for TB-T2DM patients needs to be personalized to lower resistance rates and to have better outcomes (33, 34).

Additionally, the probabilities of transitions, comparing all the times studied, led to identify the miR let-7e-5p in both the MDR-TB and MDR-TB-T2DM(-) groups, with higher transition probability at the more distant T(6-8) and T(9-12) time points, thus supporting its use as a potential biomarker.

Despite the advantages of biomarkers as non-invasive diagnostic tools, circulatory miRNAs have been reported to be redundant to similar miRNAs in various diseases and to be influenced by external factors such as smoking, diet, circadian cycles, etc. This makes difficult to link circulatory miRNAs to specific diseases (35). For these reasons, we evaluated a second *M. tuberculosis-*derived marker of the presence of proteins, DNA and RNA sequences in the serum exosomes from MDR-TB patients. Thus, sequences were amplified for 30kDa and 5KST antigens of *M. tuberculosis* from exosomal-RNA at T(9-15) after the intensive phase of treatment. A concordance between the expression of miR-let-7e-5p and the *M. tuberculosis* antigens was observed in the exosomes of 60% of patients after the intensive phase of treatment. When the expression of miR-let7e-5p decreases, the RNA for either 30kDa or 5KST proteins are detected.

A limitation of this study lies on the heterogeneity of the patients, the reduced number of DR-TB patients, and the failure to detect a miRNA associated with T2DM. Nevertheless, the results may be useful in the non-diabetic population.

## Conclusion

A dual marker presents in serum exosomes, consisting of host-derived miR-let7e-5p and *M. tuberculosis*-derived RNA for 30kDa or 5KST, may detect treatment failure or relapse for MDR-TB patients. We propose this double evaluation as a method of a long-term monitoring of TB treatment. Given the lack of methods available for detection of drug failure or relapse, these tests could help clinicians to make adjustments to therapeutic regimens and improve the control of TB in their patients.

## Data Availability Statement

The original contributions presented in the study are included in the article/**Supplementary Material**. Further inquiries can be directed to the corresponding author.

## Ethics Statement

The Ethics Committee from the Instituto Nacional de Enfermedades Respiratorias Ismael Cosío Villegas approved this study (No. C57-17). The patients/participants provided their written informed consent to participate in this study.

## Author Contributions

CC, MTH, MS-C, and YG made sample assays and data analysis. IS-G, ES, and SG-B analyzed and interpreted the data. LC-G analyzed the results. LHG-G and YG designed the study and wrote the manuscript. All authors contributed to the article and approved the submitted version.

## Funding

Fondo Sectorial de Investigación en Salud y Seguridad Social (FOSISS), Proyecto No. 00000000290282, Consejo Nacional de Ciencia y Tecnología (CONACyT), Mexico.

## Conflict of Interest

The authors declare that the research was conducted in the absence of any commercial or financial relationships that could be construed as a potential conflict of interest.

## Publisher’s Note

All claims expressed in this article are solely those of the authors and do not necessarily represent those of their affiliated organizations, or those of the publisher, the editors and the reviewers. Any product that may be evaluated in this article, or claim that may be made by its manufacturer, is not guaranteed or endorsed by the publisher.
