# Supplementary material for: A Dual Marker for Monitoring MDR-TB Treatment: Host-Derived miRNAs and M. tuberculosis-Derived RNA Sequences in Serum
Source: Front Immunol. 2021 Nov 4;12:760468. doi: 10.3389/fimmu.2021.760468 (PMC8600136; doi:10.3389/fimmu.2021.760468)
Supplement: Supplementary file 1 [file DataSheet_1.pdf]

Supplementary Material

1. The C_T value of 32 endogenous miRNAs evaluated at T(0) and T(12)

**PLATE 1**	ID	**miRNA**	**C_T (TO)**	**C_T (T12)**
A1	477860_mir	**hsa-miR-16-5p**	28.04980659	26.50959587
A2	478575_mir	**hsa-let-7a-5p**	30.23466873	28.90431595
A3	478221_mir	**hsa-let-7b-3p**	32.71641159	31.45339203
A4	478576_mir	**hsa-let-7b-5p**	34.08200455	31.86747932
A5	478577_mir	**hsa-let-7c-5p**	Undetermined	32.85919189
A6	477848_mir	**hsa-let-7d-3p**	Undetermined	29.93468475
A7	478439_mir	**hsa-let-7d-5p**	32.24302673	32.57905197
A8	478579_mir	**hsa-let-7e-5p**	Undetermined	28.68997765
A9	478578_mir	**hsa-let-7f-5p**	32.30392075	30.90026665
A10	478580_mir	**hsa-let-7g-5p**	32.25228882	30.12475967
A11	477862_mir	**hsa-let-7i-3p**	34.33986282	33.7143631
A12	478375_mir	**hsa-let-7i-5p**	29.83022499	30.43766022
B1	477820_mir	**hsa-miR-1-3p**	28.52332115	26.75643158
B2	477863_mir	**hsa-miR-101-3p**	31.71281242	29.34156227
B3	478253_mir	**hsa-miR-103a-3p**	32.35660553	32.65026093
B4	478225_mir	**hsa-miR-106a-5p**	Undetermined	Undetermined
B5	477866_mir	**hsa-miR-106b-3p**	Undetermined	38.74437714

B6	478412_mir	**hsa-miR-106b-5p**	Undetermined	29.01012421
B7	478254_mir	**hsa-miR-107**	34.0449295	33.58921814
B8	479241_mir	**hsa-miR-10a-5p**	34.46384048	31.45865822
B9	478494_mir	**hsa-miR-10b-5p**	32.19300461	32.58842087
B10	477855_mir	**hsa-miR-122-5p**	32.25159073	30.13818359
B11	477884_mir	**hsa-miR-125a-5p**	30.86580849	29.67758179
B12	477885_mir	**hsa-miR-125b-5p**	28.57684898	30.17674828
C1	477887_mir	**hsa-miR-126-3p**	27.9211731	27.3238678
C2	477889_mir	**hsa-miR-127-3p**	Undetermined	Undetermined
C3	477892_mir	**hsa-miR-128-3p**	32.40904617	32.24991608
C4	477851_mir	**hsa-miR-130a-3p**	32.07991791	31.8061924
C5	477840_mir	**hsa-miR-130b-3p**	Undetermined	Undetermined
C6	477900_mir	**hsa-miR-132-3p**	Undetermined	32.49689865
C7	478511_mir	**hsa-miR-133a-3p**	Undetermined	39.0778656
C8	480871_mir	**hsa-miR-133b**	Undetermined	39.36637115
C9	478307_mir	**hsa-miR-136-5p**	Undetermined	Undetermined
C10	478312_mir	**hsa-miR-139-5p**	31.50261879	30.35593224
C11	477908_mir	**hsa-miR-140-3p**	33.10882568	33.57636261
C12	477909_mir	**hsa-miR-140-5p**	Undetermined	31.84666634
D1	478501_mir	**hsa-miR-141-3p**	Undetermined	Undetermined
D2	477910_mir	**hsa-miR-142-3p**	30.31755257	28.44344711
D3	477911_mir	**hsa-miR-142-5p**	Undetermined	29.97034836

D4	477912_mir	**hsa-miR-143-3p**	31.26897621	30.53614235
D5	477913_mir	**hsa-miR-144-3p**	26.41192818	23.88800621
D6	477914_mir	**hsa-miR-144-5p**	Undetermined	31.07780266
D7	477916_mir	**hsa-miR-145-5p**	Undetermined	28.73421097
D8	478399_mir	**hsa-miR-146a-5p**	28.52252769	27.58912468
D9	478513_mir	**hsa-miR-146b-5p**	Undetermined	Undetermined
D10	477814_mir	**hsa-miR-148a-3p**	31.11049271	32.21821213
D11	477824_mir	**hsa-miR-148b-3p**	32.5416832	31.71259499
D12	477918_mir	**hsa-miR-150-5p**	27.15849495	27.30678368
E1	477919_mir	**hsa-miR-151a-3p**	31.65749931	33.51758194
E2	478505_mir	**hsa-miR-151a-5p**	32.98652649	31.01762009
E3	477921_mir	**hsa-miR-152-3p**	Undetermined	31.51879692
E4	477925_mir	**hsa-miR-154-5p**	Undetermined	Undetermined
E5	477927_mir	**hsa-miR-155-5p**	36.60807419	Undetermined
E6	477858_mir	**hsa-miR-15a-5p**	29.92562103	27.49378204
E7	477929_mir	**hsa-miR-15b-3p**	Undetermined	Undetermined
E8	478313_mir	**hsa-miR-15b-5p**	28.71093941	27.10866737
E9	477931_mir	**hsa-miR-16-2-3p**	33.36393738	32.87292862
E10	478447_mir	**hsa-miR-17-5p**	29.36426735	27.93149948
E11	478411_mir	**ath-miR159a**	29.20478821	30.32966423
E12	477857_mir	**hsa-miR-181a-5p**	29.77189445	29.34418678
F1	477935_mir	**hsa-miR-182-5p**	Undetermined	Undetermined
F2	477939_mir	**hsa-miR-185-5p**	30.46379471	30.66148376

F3	477940_mir	**hsa-miR-186-5p**	33.61295319	31.654459
F4	477944_mir	**hsa-miR-18a-3p**	36.9539299	Undetermined
F5	478551_mir	**hsa-miR-18a-5p**	33.0226593	32.60070038
F6	478584_mir	**hsa-miR-18b-5p**	35.00074387	34.13450623
F7	478358_mir	**hsa-miR-190a-5p**	38.07269287	36.1680069
F8	477952_mir	**hsa-miR-191-5p**	29.88350296	29.10382652
F9	478262_mir	**hsa-miR-192-5p**	28.84110832	29.39526558
F10	478314_mir	**hsa-miR-193b-3p**	Undetermined	31.2579689
F11	477956_mir	**hsa-miR-194-5p**	33.23752594	32.20273209
F12	477957_mir	**hsa-miR-195-5p**	31.60767365	Undetermined
G1	477959_mir	**hsa-miR-197-3p**	Undetermined	24.18584442
G2	477961_mir	**hsa-miR-199a-3p_hsa-miR-199b-3p**	29.94068336	28.73979568
G3	478231_mir	**hsa-miR-199a-5p**	Undetermined	31.90057564
G4	479228_mir	**hsa-miR-19a-3p**	32.55803299	30.88954735
G5	478264_mir	**hsa-miR-19b-3p**	32.69347763	31.01452827
G6	478490_mir	**hsa-miR-200a-3p**	34.42556381	33.70599365
G7	478351_mir	**hsa-miR-200c-3p**	34.90829468	34.592556
G8	478491_mir	**hsa-miR-204-5p**	37.10448837	38.22386932
G9	477967_mir	**hsa-miR-205-5p**	27.96895599	28.09300613
G10	477819_mir	**hsa-miR-208a-3p**	Undetermined	Undetermined
G11	478317_mir	**hsa-miR-20a-3p**	29.05567551	Undetermined
G12	478586_mir	**hsa-miR-20a-5p**	28.69412422	28.2353878

H1	477804_mir	**hsa-miR-20b-5p**	Undetermined	31.00663376
H2	477973_mir	**hsa-miR-21-3p**	Undetermined	34.49645233
H3	477975_mir	**hsa-miR-21-5p**	27.98845291	27.52995491
H4	477970_mir	**hsa-miR-210-3p**	34.12370682	32.45740128
H5	477971_mir	**hsa-miR-2110**	37.86182022	36.23454666
H6	478516_mir	**hsa-miR-215-5p**	Undetermined	Undetermined
H7	477985_mir	**hsa-miR-22-3p**	28.42376709	28.14249229
H8	477987_mir	**hsa-miR-22-5p**	Undetermined	33.36706924
H9	477981_mir	**hsa-miR-221-3p**	27.92764473	27.34396172
H10	477982_mir	**hsa-miR-222-3p**	34.298172	34.84630966
H11	477983_mir	**hsa-miR-223-3p**	25.41051483	25.47878075
H12	477984_mir	**hsa-miR-223-5p**	Undetermined	Undetermined

**PLATE 2**	ID	**miRNA**	**C_T (TO)**	**C_T (T12)**
A1	477860_mir	**hsa-miR-16-5p**	27.77269173	26.43927193
A2	477986_mir	**hsa-miR-224-5p**	32.59539413	32.74313354
A3	478532_mir	**hsa-miR-23a-3p**	29.72961235	30.02237892
A4	478602_mir	**hsa-miR-23b-3p**	31.22785568	30.95518494
A5	477992_mir	**hsa-miR-24-3p**	29.69152451	28.03481102
A6	477994_mir	**hsa-miR-25-3p**	28.64697266	27.82489777
A7	477995_mir	**hsa-miR-26a-5p**	27.81258774	27.3546505
A8	478418_mir	**hsa-miR-26b-5p**	27.35288239	26.82007408
A9	478384_mir	**hsa-miR-27a-3p**	29.97758675	29.907938

A10	478270_mir	**hsa-miR-27b-3p**	31.96850395	32.11865616
A11	477999_mir	**hsa-miR-28-3p**	35.77980423	33.64974213
A12	478293_mir	**cel-miR-39-3p**	Undetermined	Undetermined
B1	478000_mir	**hsa-miR-28-5p**	34.04742813	35.47568512
B2	477836_mir	**hsa-miR-296-5p**	Undetermined	Undetermined
B3	478587_mir	**hsa-miR-29a-3p**	30.67967224	31.33153343
B4	478002_mir	**hsa-miR-29a-5p**	29.01394272	28.70182228
B5	478003_mir	**hsa-miR-29b-2-5p**	Undetermined	Undetermined
B6	478369_mir	**hsa-miR-29b-3p**	32.03409195	31.32423401
B7	479229_mir	**hsa-miR-29c-3p**	Undetermined	31.01079178
B8	477815_mir	**hsa-miR-301a-3p**	Undetermined	Undetermined
B9	477825_mir	**hsa-miR-301b-3p**	Undetermined	Undetermined
B10	479448_mir	**hsa-miR-30a-5p**	Undetermined	Undetermined
B11	478007_mir	**hsa-miR-30b-5p**	29.19677353	28.88189888
B12	478008_mir	**hsa-miR-30c-5p**	Undetermined	28.90625572
C1	478606_mir	**hsa-miR-30d-5p**	35.02662659	34.47704315
C2	478388_mir	**hsa-miR-30e-3p**	Undetermined	Undetermined
C3	479235_mir	**hsa-miR-30e-5p**	Undetermined	30.7750473
C4	478015_mir	**hsa-miR-31-5p**	35.08982849	36.20032501
C5	478026_mir	**hsa-miR-32-5p**	Undetermined	Undetermined
C6	478594_mir	**hsa-miR-320a**	28.69087219	29.31896019
C7	478588_mir	**hsa-miR-320b**	30.86668587	31.12192535

C8	478022_mir	**hsa-miR-320e**	28.81337166	28.89036179
C9	478023_mir	**hsa-miR-324-3p**	Undetermined	29.7976265
C10	478024_mir	**hsa-miR-324-5p**	31.36130714	30.15415192
C11	478027_mir	**hsa-miR-326**	36.87876511	31.31696892
C12	478028_mir	**hsa-miR-328-3p**	32.32848358	Undetermined
D1	478323_mir	**hsa-miR-331-3p**	Undetermined	31.75607491
D2	478324_mir	**hsa-miR-335-5p**	Undetermined	Undetermined
D3	478037_mir	**hsa-miR-338-3p**	Undetermined	34.54976273
D4	478325_mir	**hsa-miR-339-3p**	35.00676727	34.32398987
D5	478040_mir	**hsa-miR-339-5p**	32.93803024	33.29735565
D6	478347_mir	**hsa-miR-33a-5p**	Undetermined	33.20317841
D7	478043_mir	**hsa-miR-342-3p**	31.91996002	30.93192863
D8	478046_mir	**hsa-miR-346**	Undetermined	Undetermined
D9	478048_mir	**hsa-miR-34a-5p**	Undetermined	Undetermined
D10	478055_mir	**hsa-miR-361-3p**	33.16503143	37.48524475
D11	478060_mir	**hsa-miR-363-3p**	Undetermined	32.62764359
D12	478065_mir	**hsa-miR-365a-3p**	Undetermined	Undetermined
E1	478363_mir	**hsa-miR-373-3p**	35.99824142	36.32544708
E2	478073_mir	**hsa-miR-373-5p**	Undetermined	36.82288742
E3	478238_mir	**hsa-miR-374a-5p**	34.54343414	33.97453308
E4	478389_mir	**hsa-miR-374b-5p**	33.54689407	32.9817276
E5	478074_mir	**hsa-miR-375**	25.49435997	27.4288311
E6	478240_mir	**hsa-miR-376a-3p**	32.20997238	33.58529663

E7	478349_mir	**hsa-miR-378a-3p**	33.4217186	33.54700851
E8	478078_mir	**hsa-miR-382-5p**	Undetermined	Undetermined
E9	478084_mir	**hsa-miR-409-3p**	Undetermined	39.14609909
E10	478088_mir	**hsa-miR-421**	Undetermined	Undetermined
E11	478327_mir	**hsa-miR-423-3p**	31.59069633	31.04014778
E12	478090_mir	**hsa-miR-423-5p**	29.00276375	28.07009315
F1	478092_mir	**hsa-miR-424-5p**	32.49365997	30.70918846
F2	478093_mir	**hsa-miR-425-3p**	Undetermined	Undetermined
F3	478094_mir	**hsa-miR-425-5p**	31.19877052	30.31438828
F4	478107_mir	**hsa-miR-451a**	21.85524368	21.67033005
F5	478122_mir	**hsa-miR-483-3p**	31.12688446	33.36904144
F6	478432_mir	**hsa-miR-483-5p**	32.038517	32.43738937
F7	478308_mir	**hsa-miR-484**	30.569664	30.0887661
F8	478125_mir	**hsa-miR-485-3p**	Undetermined	Undetermined
F9	478128_mir	**hsa-miR-486-5p**	28.55716133	27.15333748
F10	478136_mir	**hsa-miR-495-3p**	37.64415359	36.4057579
F11	478138_mir	**hsa-miR-497-5p**	Undetermined	Undetermined
F12	478309_mir	**hsa-miR-500a-5p**	Undetermined	Undetermined
G1	478350_mir	**hsa-miR-501-3p**	31.11302757	30.74193764
G2	478348_mir	**hsa-miR-502-3p**	29.84033394	30.41969872
G3	478145_mir	**hsa-miR-505-3p**	34.9071312	33.83415604
G4	478336_mir	**hsa-miR-532-3p**	28.20897675	29.07426071

G5	478151_mir	**hsa-miR-532-5p**	Undetermined	Undetermined
G6	478155_mir	**hsa-miR-543**	33.52133942	Undetermined
G7	478158_mir	**hsa-miR-551a**	30.58221817	31.96788979
G8	478159_mir	**hsa-miR-551b-3p**	Undetermined	31.47094536
G9	478163_mir	**hsa-miR-574-3p**	Undetermined	Undetermined
G10	478167_mir	**hsa-miR-584-5p**	26.34005165	27.03897667
G11	478367_mir	**hsa-miR-590-5p**	Undetermined	32.80963135
G12	478174_mir	**hsa-miR-605-5p**	Undetermined	Undetermined
H1	478183_mir	**hsa-miR-629-5p**	Undetermined	Undetermined
H2	478189_mir	**hsa-miR-652-3p**	29.93979836	28.24447632
H3	478192_mir	**hsa-miR-660-5p**	34.07609558	33.51945877
H4	478342_mir	**hsa-miR-766-3p**	Undetermined	Undetermined
H5	478207_mir	**hsa-miR-885-5p**	Undetermined	33.82443237
H6	477827_mir	**hsa-miR-92a-3p**	26.7600708	26.82304192
H7	477823_mir	**hsa-miR-92b-3p**	27.56397057	27.94299126
H8	478209_mir	**hsa-miR-93-3p**	Undetermined	35.20497131
H9	478210_mir	**hsa-miR-93-5p**	30.66248894	30.32363701
H10	478213_mir	**hsa-miR-95-3p**	Undetermined	Undetermined
H11	478519_mir	**hsa-miR-99a-5p**	Undetermined	Undetermined
H12	478343_mir	**hsa-miR-99b-5p**	33.22515488	35.08739853

2. The C_T value of 180 unique miRNAs evaluated at T(0) and T(12)

**Plate 1**			**T(0)**
**Well**	**ID**	**miRNA**	**C_T**
A2	478575_mir	**hsa-let-7a-5p**	30.23466873
A3	478221_mir	**hsa-let-7b-3p**	32.71641159
A4	478576_mir	**hsa-let-7b-5p**	34.08200455
A5	478577_mir	**hsa-let-7c-5p**	Undetermined
A6	477848_mir	**hsa-let-7d-3p**	Undetermined
A7	478439_mir	**hsa-let-7d-5p**	32.24302673
A8	478579_mir	**hsa-let-7e-5p**	Undetermined
A9	478578_mir	**hsa-let-7f-5p**	32.30392075
A10	478580_mir	**hsa-let-7g-5p**	32.25228882
A11	477862_mir	**hsa-let-7i-3p**	34.33986282
A12	478375_mir	**hsa-let-7i-5p**	29.83022499
B1	477820_mir	**hsa-miR-1-3p**	28.52332115
B2	477863_mir	**hsa-miR-101-3p**	31.71281242
B3	478253_mir	**hsa-miR-103a-3p**	32.35660553
B4	478225_mir	**hsa-miR-106a-5p**	Undetermined
B5	477866_mir	**hsa-miR-106b-3p**	Undetermined
B6	478412_mir	**hsa-miR-106b-5p**	Undetermined

B7	478254_mir	**hsa-miR-107**	34.0449295
B8	479241_mir	**hsa-miR-10a-5p**	34.46384048
B9	478494_mir	**hsa-miR-10b-5p**	32.19300461
B10	477855_mir	**hsa-miR-122-5p**	32.25159073
B11	477884_mir	**hsa-miR-125a-5p**	30.86580849
B12	477885_mir	**hsa-miR-125b-5p**	28.57684898
C1	477887_mir	**hsa-miR-126-3p**	27.9211731
C2	477889_mir	**hsa-miR-127-3p**	Undetermined
C3	477892_mir	**hsa-miR-128-3p**	32.40904617
C4	477851_mir	**hsa-miR-130a-3p**	32.07991791
C5	477840_mir	**hsa-miR-130b-3p**	Undetermined
C6	477900_mir	**hsa-miR-132-3p**	Undetermined
C7	478511_mir	**hsa-miR-133a-3p**	Undetermined
C8	480871_mir	**hsa-miR-133b**	Undetermined
C9	478307_mir	**hsa-miR-136-5p**	Undetermined
C10	478312_mir	**hsa-miR-139-5p**	31.50261879
C11	477908_mir	**hsa-miR-140-3p**	33.10882568
C12	477909_mir	**hsa-miR-140-5p**	Undetermined
D1	478501_mir	**hsa-miR-141-3p**	Undetermined
D2	477910_mir	**hsa-miR-142-3p**	30.31755257
D3	477911_mir	**hsa-miR-142-5p**	Undetermined
D4	477912_mir	**hsa-miR-143-3p**	31.26897621
D5	477913_mir	**hsa-miR-144-3p**	26.41192818

D6	477914_mir	**hsa-miR-144-5p**	Undetermined
D7	477916_mir	**hsa-miR-145-5p**	Undetermined
D8	478399_mir	**hsa-miR-146a-5p**	28.52252769
D9	478513_mir	**hsa-miR-146b-5p**	Undetermined
D10	477814_mir	**hsa-miR-148a-3p**	31.11049271
D11	477824_mir	**hsa-miR-148b-3p**	32.5416832
D12	477918_mir	**hsa-miR-150-5p**	27.15849495
E1	477919_mir	**hsa-miR-151a-3p**	31.65749931
E2	478505_mir	**hsa-miR-151a-5p**	32.98652649
E3	477921_mir	**hsa-miR-152-3p**	Undetermined
E4	477925_mir	**hsa-miR-154-5p**	Undetermined
E5	477927_mir	**hsa-miR-155-5p**	36.60807419
E6	477858_mir	**hsa-miR-15a-5p**	29.92562103
E7	477929_mir	**hsa-miR-15b-3p**	Undetermined
E8	478313_mir	**hsa-miR-15b-5p**	28.71093941
E9	477931_mir	**hsa-miR-16-2-3p**	33.36393738
E10	478447_mir	**hsa-miR-17-5p**	29.36426735
E12	477857_mir	**hsa-miR-181a-5p**	29.77189445
F1	477935_mir	**hsa-miR-182-5p**	Undetermined
F2	477939_mir	**hsa-miR-185-5p**	30.46379471
F3	477940_mir	**hsa-miR-186-5p**	33.61295319
F4	477944_mir	**hsa-miR-18a-3p**	36.9539299

F5	478551_mir	**hsa-miR-18a-5p**	33.0226593
F6	478584_mir	**hsa-miR-18b-5p**	35.00074387
F7	478358_mir	**hsa-miR-190a-5p**	38.07269287
F8	477952_mir	**hsa-miR-191-5p**	29.88350296
F9	478262_mir	**hsa-miR-192-5p**	28.84110832
F10	478314_mir	**hsa-miR-193b-3p**	Undetermined
F11	477956_mir	**hsa-miR-194-5p**	33.23752594
F12	477957_mir	**hsa-miR-195-5p**	31.60767365
G1	477959_mir	**hsa-miR-197-3p**	Undetermined
G2	477961_mir	**hsa-miR-199a-3p_hsa-miR-199b-3p**	29.94068336
G3	478231_mir	**hsa-miR-199a-5p**	Undetermined
G4	479228_mir	**hsa-miR-19a-3p**	32.55803299
G5	478264_mir	**hsa-miR-19b-3p**	32.69347763
G6	478490_mir	**hsa-miR-200a-3p**	34.42556381
G7	478351_mir	**hsa-miR-200c-3p**	34.90829468
G8	478491_mir	**hsa-miR-204-5p**	37.10448837
G9	477967_mir	**hsa-miR-205-5p**	27.96895599
G10	477819_mir	**hsa-miR-208a-3p**	Undetermined
G11	478317_mir	**hsa-miR-20a-3p**	29.05567551
G12	478586_mir	**hsa-miR-20a-5p**	28.69412422
H1	477804_mir	**hsa-miR-20b-5p**	Undetermined
H2	477973_mir	**hsa-miR-21-3p**	Undetermined

H3	477975_mir	**hsa-miR-21-5p**	27.98845291
H4	477970_mir	**hsa-miR-210-3p**	34.12370682
H5	477971_mir	**hsa-miR-2110**	37.86182022
H6	478516_mir	**hsa-miR-215-5p**	Undetermined
H7	477985_mir	**hsa-miR-22-3p**	28.42376709
H8	477987_mir	**hsa-miR-22-5p**	Undetermined
H9	477981_mir	**hsa-miR-221-3p**	27.92764473
H10	477982_mir	**hsa-miR-222-3p**	34.298172
H11	477983_mir	**hsa-miR-223-3p**	25.41051483
H12	477984_mir	**hsa-miR-223-5p**	Undetermined
**Plate 2**			**T(0)**
**Well**	**ID**	**miRNA**	**C_T**
A2	477986_mir	**hsa-miR-224-5p**	32.59539413
A3	478532_mir	**hsa-miR-23a-3p**	29.72961235
A4	478602_mir	**hsa-miR-23b-3p**	31.22785568
A5	477992_mir	**hsa-miR-24-3p**	29.69152451
A6	477994_mir	**hsa-miR-25-3p**	28.64697266
A7	477995_mir	**hsa-miR-26a-5p**	27.81258774
A8	478418_mir	**hsa-miR-26b-5p**	27.35288239
A9	478384_mir	**hsa-miR-27a-3p**	29.97758675
A10	478270_mir	**hsa-miR-27b-3p**	31.96850395
A11	477999_mir	**hsa-miR-28-3p**	35.77980423

B1	478000_mir	**hsa-miR-28-5p**	34.04742813
B2	477836_mir	**hsa-miR-296-5p**	Undetermined
B3	478587_mir	**hsa-miR-29a-3p**	30.67967224
B4	478002_mir	**hsa-miR-29a-5p**	29.01394272
B5	478003_mir	**hsa-miR-29b-2-5p**	Undetermined
B6	478369_mir	**hsa-miR-29b-3p**	32.03409195
B7	479229_mir	**hsa-miR-29c-3p**	Undetermined
B8	477815_mir	**hsa-miR-301a-3p**	Undetermined
B9	477825_mir	**hsa-miR-301b-3p**	Undetermined
B10	479448_mir	**hsa-miR-30a-5p**	Undetermined
B11	478007_mir	**hsa-miR-30b-5p**	29.19677353
B12	478008_mir	**hsa-miR-30c-5p**	Undetermined
C1	478606_mir	**hsa-miR-30d-5p**	35.02662659
C2	478388_mir	**hsa-miR-30e-3p**	Undetermined
C3	479235_mir	**hsa-miR-30e-5p**	Undetermined
C4	478015_mir	**hsa-miR-31-5p**	35.08982849
C5	478026_mir	**hsa-miR-32-5p**	Undetermined
C6	478594_mir	**hsa-miR-320a**	28.69087219
C7	478588_mir	**hsa-miR-320b**	30.86668587
C8	478022_mir	**hsa-miR-320e**	28.81337166
C9	478023_mir	**hsa-miR-324-3p**	Undetermined
C10	478024_mir	**hsa-miR-324-5p**	31.36130714
C11	478027_mir	**hsa-miR-326**	36.87876511

C12	478028_mir	**hsa-miR-328-3p**	32.32848358
D1	478323_mir	**hsa-miR-331-3p**	Undetermined
D2	478324_mir	**hsa-miR-335-5p**	Undetermined
D3	478037_mir	**hsa-miR-338-3p**	Undetermined
D4	478325_mir	**hsa-miR-339-3p**	35.00676727
D5	478040_mir	**hsa-miR-339-5p**	32.93803024
D6	478347_mir	**hsa-miR-33a-5p**	Undetermined
D7	478043_mir	**hsa-miR-342-3p**	31.91996002
D8	478046_mir	**hsa-miR-346**	Undetermined
D9	478048_mir	**hsa-miR-34a-5p**	Undetermined
D10	478055_mir	**hsa-miR-361-3p**	33.16503143
D11	478060_mir	**hsa-miR-363-3p**	Undetermined
D12	478065_mir	**hsa-miR-365a-3p_hsa-miR-365b-3p**	Undetermined
E1	478363_mir	**hsa-miR-373-3p**	35.99824142
E2	478073_mir	**hsa-miR-373-5p**	Undetermined
E3	478238_mir	**hsa-miR-374a-5p**	34.54343414
E4	478389_mir	**hsa-miR-374b-5p**	33.54689407
E5	478074_mir	**hsa-miR-375**	25.49435997
E6	478240_mir	**hsa-miR-376a-3p**	32.20997238
E7	478349_mir	**hsa-miR-378a-3p**	33.4217186
E8	478078_mir	**hsa-miR-382-5p**	Undetermined
E9	478084_mir	**hsa-miR-409-3p**	Undetermined

E10	478088_mir	**hsa-miR-421**	Undetermined
E11	478327_mir	**hsa-miR-423-3p**	31.59069633
E12	478090_mir	**hsa-miR-423-5p**	29.00276375
F1	478092_mir	**hsa-miR-424-5p**	32.49365997
F2	478093_mir	**hsa-miR-425-3p**	Undetermined
F3	478094_mir	**hsa-miR-425-5p**	31.19877052
F4	478107_mir	**hsa-miR-451a**	21.85524368
F5	478122_mir	**hsa-miR-483-3p**	31.12688446
F6	478432_mir	**hsa-miR-483-5p**	32.038517
F7	478308_mir	**hsa-miR-484**	30.569664
F8	478125_mir	**hsa-miR-485-3p**	Undetermined
F9	478128_mir	**hsa-miR-486-5p**	28.55716133
F10	478136_mir	**hsa-miR-495-3p**	37.64415359
F11	478138_mir	**hsa-miR-497-5p**	Undetermined
F12	478309_mir	**hsa-miR-500a-5p**	Undetermined
G1	478350_mir	**hsa-miR-501-3p**	31.11302757
G2	478348_mir	**hsa-miR-502-3p**	29.84033394
G3	478145_mir	**hsa-miR-505-3p**	34.9071312
G4	478336_mir	**hsa-miR-532-3p**	28.20897675
G5	478151_mir	**hsa-miR-532-5p**	Undetermined
G6	478155_mir	**hsa-miR-543**	33.52133942
G7	478158_mir	**hsa-miR-551a**	30.58221817
G8	478159_mir	**hsa-miR-551b-3p**	Undetermined

G9	478163_mir	**hsa-miR-574-3p**	Undetermined
G10	478167_mir	**hsa-miR-584-5p**	26.34005165
G11	478367_mir	**hsa-miR-590-5p**	Undetermined
G12	478174_mir	**hsa-miR-605-5p**	Undetermined
H1	478183_mir	**hsa-miR-629-5p**	Undetermined
H2	478189_mir	**hsa-miR-652-3p**	29.93979836
H3	478192_mir	**hsa-miR-660-5p**	34.07609558
H4	478342_mir	**hsa-miR-766-3p**	Undetermined
H5	478207_mir	**hsa-miR-885-5p**	Undetermined
H6	477827_mir	**hsa-miR-92a-3p**	26.7600708
H7	477823_mir	**hsa-miR-92b-3p**	27.56397057
H8	478209_mir	**hsa-miR-93-3p**	Undetermined
H9	478210_mir	**hsa-miR-93-5p**	30.66248894
H10	478213_mir	**hsa-miR-95-3p**	Undetermined
H11	478519_mir	**hsa-miR-99a-5p**	Undetermined
H12	478343_mir	**hsa-miR-99b-5p**	33.22515488

**Plate 1**			**T(12)**
**Well**	**ID**	**miRNA**	**C_T**
A2	478575_mir	**hsa-let-7a-5p**	28.90431595
A3	478221_mir	**hsa-let-7b-3p**	31.45339203

A4	478576_mir	**hsa-let-7b-5p**	31.86747932
A5	478577_mir	**hsa-let-7c-5p**	32.85919189
A6	477848_mir	**hsa-let-7d-3p**	29.93468475
A7	478439_mir	**hsa-let-7d-5p**	32.57905197
A8	478579_mir	**hsa-let-7e-5p**	28.68997765
A9	478578_mir	**hsa-let-7f-5p**	30.90026665
A10	478580_mir	**hsa-let-7g-5p**	30.12475967
A11	477862_mir	**hsa-let-7i-3p**	33.7143631
A12	478375_mir	**hsa-let-7i-5p**	30.43766022
B1	477820_mir	**hsa-miR-1-3p**	26.75643158
B2	477863_mir	**hsa-miR-101-3p**	29.34156227
B3	478253_mir	**hsa-miR-103a-3p**	32.65026093
B4	478225_mir	**hsa-miR-106a-5p**	Undetermined
B5	477866_mir	**hsa-miR-106b-3p**	38.74437714
B6	478412_mir	**hsa-miR-106b-5p**	29.01012421
B7	478254_mir	**hsa-miR-107**	33.58921814
B8	479241_mir	**hsa-miR-10a-5p**	31.45865822
B9	478494_mir	**hsa-miR-10b-5p**	32.58842087
B10	477855_mir	**hsa-miR-122-5p**	30.13818359
B11	477884_mir	**hsa-miR-125a-5p**	29.67758179
B12	477885_mir	**hsa-miR-125b-5p**	30.17674828
C1	477887_mir	**hsa-miR-126-3p**	27.3238678
C2	477889_mir	**hsa-miR-127-3p**	Undetermined

C3	477892_mir	**hsa-miR-128-3p**	32.24991608
C4	477851_mir	**hsa-miR-130a-3p**	31.8061924
C5	477840_mir	**hsa-miR-130b-3p**	Undetermined
C6	477900_mir	**hsa-miR-132-3p**	32.49689865
C7	478511_mir	**hsa-miR-133a-3p**	39.0778656
C8	480871_mir	**hsa-miR-133b**	39.36637115
C9	478307_mir	**hsa-miR-136-5p**	Undetermined
C10	478312_mir	**hsa-miR-139-5p**	30.35593224
C11	477908_mir	**hsa-miR-140-3p**	33.57636261
C12	477909_mir	**hsa-miR-140-5p**	31.84666634
D1	478501_mir	**hsa-miR-141-3p**	Undetermined
D2	477910_mir	**hsa-miR-142-3p**	28.44344711
D3	477911_mir	**hsa-miR-142-5p**	29.97034836
D4	477912_mir	**hsa-miR-143-3p**	30.53614235
D5	477913_mir	**hsa-miR-144-3p**	23.88800621
D6	477914_mir	**hsa-miR-144-5p**	31.07780266
D7	477916_mir	**hsa-miR-145-5p**	28.73421097
D8	478399_mir	**hsa-miR-146a-5p**	27.58912468
D9	478513_mir	**hsa-miR-146b-5p**	Undetermined
D10	477814_mir	**hsa-miR-148a-3p**	32.21821213
D11	477824_mir	**hsa-miR-148b-3p**	31.71259499
D12	477918_mir	**hsa-miR-150-5p**	27.30678368

E1	477919_mir	**hsa-miR-151a-3p**	33.51758194
E2	478505_mir	**hsa-miR-151a-5p**	31.01762009
E3	477921_mir	**hsa-miR-152-3p**	31.51879692
E4	477925_mir	**hsa-miR-154-5p**	Undetermined
E5	477927_mir	**hsa-miR-155-5p**	Undetermined
E6	477858_mir	**hsa-miR-15a-5p**	27.49378204
E7	477929_mir	**hsa-miR-15b-3p**	Undetermined
E8	478313_mir	**hsa-miR-15b-5p**	27.10866737
E9	477931_mir	**hsa-miR-16-2-3p**	32.87292862
E10	478447_mir	**hsa-miR-17-5p**	27.93149948
E12	477857_mir	**hsa-miR-181a-5p**	29.34418678
F1	477935_mir	**hsa-miR-182-5p**	Undetermined
F2	477939_mir	**hsa-miR-185-5p**	30.66148376
F3	477940_mir	**hsa-miR-186-5p**	31.654459
F4	477944_mir	**hsa-miR-18a-3p**	Undetermined
F5	478551_mir	**hsa-miR-18a-5p**	32.60070038
F6	478584_mir	**hsa-miR-18b-5p**	34.13450623
F7	478358_mir	**hsa-miR-190a-5p**	36.1680069
F8	477952_mir	**hsa-miR-191-5p**	29.10382652
F9	478262_mir	**hsa-miR-192-5p**	29.39526558
F10	478314_mir	**hsa-miR-193b-3p**	31.2579689
F11	477956_mir	**hsa-miR-194-5p**	32.20273209
F12	477957_mir	**hsa-miR-195-5p**	Undetermined

G1	477959_mir	**hsa-miR-197-3p**	24.18584442
G2	477961_mir	**hsa-miR-199a-3p_hsa-miR-199b-3p**	28.73979568
G3	478231_mir	**hsa-miR-199a-5p**	31.90057564
G4	479228_mir	**hsa-miR-19a-3p**	30.88954735
G5	478264_mir	**hsa-miR-19b-3p**	31.01452827
G6	478490_mir	**hsa-miR-200a-3p**	33.70599365
G7	478351_mir	**hsa-miR-200c-3p**	34.592556
G8	478491_mir	**hsa-miR-204-5p**	38.22386932
G9	477967_mir	**hsa-miR-205-5p**	28.09300613
G10	477819_mir	**hsa-miR-208a-3p**	Undetermined
G11	478317_mir	**hsa-miR-20a-3p**	Undetermined
G12	478586_mir	**hsa-miR-20a-5p**	28.2353878
H1	477804_mir	**hsa-miR-20b-5p**	31.00663376
H2	477973_mir	**hsa-miR-21-3p**	34.49645233
H3	477975_mir	**hsa-miR-21-5p**	27.52995491
H4	477970_mir	**hsa-miR-210-3p**	32.45740128
H5	477971_mir	**hsa-miR-2110**	36.23454666
H6	478516_mir	**hsa-miR-215-5p**	Undetermined
H7	477985_mir	**hsa-miR-22-3p**	28.14249229
H8	477987_mir	**hsa-miR-22-5p**	33.36706924
H9	477981_mir	**hsa-miR-221-3p**	27.34396172
H10	477982_mir	**hsa-miR-222-3p**	34.84630966

H11	477983_mir	**hsa-miR-223-3p**	25.47878075
H12	477984_mir	**hsa-miR-223-5p**	Undetermined
**Plate 2**			**T(12)**
**Well**	**ID**	**miRNA**	**Cт**
A2	477986_mir	**hsa-miR-224-5p**	32.74313354
A3	478532_mir	**hsa-miR-23a-3p**	30.02237892
A4	478602_mir	**hsa-miR-23b-3p**	30.95518494
A5	477992_mir	**hsa-miR-24-3p**	28.03481102
A6	477994_mir	**hsa-miR-25-3p**	27.82489777
A7	477995_mir	**hsa-miR-26a-5p**	27.3546505
A8	478418_mir	**hsa-miR-26b-5p**	26.82007408
A9	478384_mir	**hsa-miR-27a-3p**	29.907938
A10	478270_mir	**hsa-miR-27b-3p**	32.11865616
A11	477999_mir	**hsa-miR-28-3p**	33.64974213
B1	478000_mir	**hsa-miR-28-5p**	35.47568512
B2	477836_mir	**hsa-miR-296-5p**	Undetermined
B3	478587_mir	**hsa-miR-29a-3p**	31.33153343
B4	478002_mir	**hsa-miR-29a-5p**	28.70182228
B5	478003_mir	**hsa-miR-29b-2-5p**	Undetermined
B6	478369_mir	**hsa-miR-29b-3p**	31.32423401
B7	479229_mir	**hsa-miR-29c-3p**	31.01079178
B8	477815_mir	**hsa-miR-301a-3p**	Undetermined
B9	477825_mir	**hsa-miR-301b-3p**	Undetermined

B10	479448_mir	**hsa-miR-30a-5p**	Undetermined
B11	478007_mir	**hsa-miR-30b-5p**	28.88189888
B12	478008_mir	**hsa-miR-30c-5p**	28.90625572
C1	478606_mir	**hsa-miR-30d-5p**	34.47704315
C2	478388_mir	**hsa-miR-30e-3p**	Undetermined
C3	479235_mir	**hsa-miR-30e-5p**	30.7750473
C4	478015_mir	**hsa-miR-31-5p**	36.20032501
C5	478026_mir	**hsa-miR-32-5p**	Undetermined
C6	478594_mir	**hsa-miR-320a**	29.31896019
C7	478588_mir	**hsa-miR-320b**	31.12192535
C8	478022_mir	**hsa-miR-320e**	28.89036179
C9	478023_mir	**hsa-miR-324-3p**	29.7976265
C10	478024_mir	**hsa-miR-324-5p**	30.15415192
C11	478027_mir	**hsa-miR-326**	31.31696892
C12	478028_mir	**hsa-miR-328-3p**	Undetermined
D1	478323_mir	**hsa-miR-331-3p**	31.75607491
D2	478324_mir	**hsa-miR-335-5p**	Undetermined
D3	478037_mir	**hsa-miR-338-3p**	34.54976273
D4	478325_mir	**hsa-miR-339-3p**	34.32398987
D5	478040_mir	**hsa-miR-339-5p**	33.29735565
D6	478347_mir	**hsa-miR-33a-5p**	33.20317841
D7	478043_mir	**hsa-miR-342-3p**	30.93192863

D8	478046_mir	**hsa-miR-346**	Undetermined
D9	478048_mir	**hsa-miR-34a-5p**	Undetermined
D10	478055_mir	**hsa-miR-361-3p**	37.48524475
D11	478060_mir	**hsa-miR-363-3p**	32.62764359
D12	478065_mir	**hsa-miR-365a-3p_hsa-miR-365b-3p**	Undetermined
E1	478363_mir	**hsa-miR-373-3p**	36.32544708
E2	478073_mir	**hsa-miR-373-5p**	36.82288742
E3	478238_mir	**hsa-miR-374a-5p**	33.97453308
E4	478389_mir	**hsa-miR-374b-5p**	32.9817276
E5	478074_mir	**hsa-miR-375**	27.4288311
E6	478240_mir	**hsa-miR-376a-3p**	33.58529663
E7	478349_mir	**hsa-miR-378a-3p**	33.54700851
E8	478078_mir	**hsa-miR-382-5p**	Undetermined
E9	478084_mir	**hsa-miR-409-3p**	39.14609909
E10	478088_mir	**hsa-miR-421**	Undetermined
E11	478327_mir	**hsa-miR-423-3p**	31.04014778
E12	478090_mir	**hsa-miR-423-5p**	28.07009315
F1	478092_mir	**hsa-miR-424-5p**	30.70918846
F2	478093_mir	**hsa-miR-425-3p**	Undetermined
F3	478094_mir	**hsa-miR-425-5p**	30.31438828
F4	478107_mir	**hsa-miR-451a**	21.67033005
F5	478122_mir	**hsa-miR-483-3p**	33.36904144

F6	478432_mir	**hsa-miR-483-5p**	32.43738937
F7	478308_mir	**hsa-miR-484**	30.0887661
F8	478125_mir	**hsa-miR-485-3p**	Undetermined
F9	478128_mir	**hsa-miR-486-5p**	27.15333748
F10	478136_mir	**hsa-miR-495-3p**	36.4057579
F11	478138_mir	**hsa-miR-497-5p**	Undetermined
F12	478309_mir	**hsa-miR-500a-5p**	Undetermined
G1	478350_mir	**hsa-miR-501-3p**	30.74193764
G2	478348_mir	**hsa-miR-502-3p**	30.41969872
G3	478145_mir	**hsa-miR-505-3p**	33.83415604
G4	478336_mir	**hsa-miR-532-3p**	29.07426071
G5	478151_mir	**hsa-miR-532-5p**	Undetermined
G6	478155_mir	**hsa-miR-543**	Undetermined
G7	478158_mir	**hsa-miR-551a**	31.96788979
G8	478159_mir	**hsa-miR-551b-3p**	31.47094536
G9	478163_mir	**hsa-miR-574-3p**	Undetermined
G10	478167_mir	**hsa-miR-584-5p**	27.03897667
G11	478367_mir	**hsa-miR-590-5p**	32.80963135
G12	478174_mir	**hsa-miR-605-5p**	Undetermined
H1	478183_mir	**hsa-miR-629-5p**	Undetermined
H2	478189_mir	**hsa-miR-652-3p**	28.24447632
H3	478192_mir	**hsa-miR-660-5p**	33.51945877

H4	478342_mir	**hsa-miR-766-3p**	Undetermined
H5	478207_mir	**hsa-miR-885-5p**	33.82443237
H6	477827_mir	**hsa-miR-92a-3p**	26.82304192
H7	477823_mir	**hsa-miR-92b-3p**	27.94299126
H8	478209_mir	**hsa-miR-93-3p**	35.20497131
H9	478210_mir	**hsa-miR-93-5p**	30.32363701
H10	478213_mir	**hsa-miR-95-3p**	Undetermined
H11	478519_mir	**hsa-miR-99a-5p**	Undetermined
H12	478343_mir	**hsa-miR-99b-5p**	35.08739853
